# Hearing impairment is common among Saami adults in Northern Finland

**DOI:** 10.1080/22423982.2017.1398004

**Published:** 2017-11-13

**Authors:** Venla Lohi, Pasi Ohtonen, Pekka Aikio, Martti Sorri, Elina Mäki-Torkko, Samuli Hannula

**Affiliations:** ^a^ PEDEGO Research Unit, University of Oulu, Oulu, Finland; ^b^ Medical Research Center Oulu, University of Oulu and Oulu University Hospital, Oulu, Finland; ^c^ Department of Otorhinolaryngology and Head and Neck Surgery, Oulu University Hospital, Oulu, Finland; ^d^ Division of Operative Care and Medical Research Center Oulu, Oulu University Hospital and University of Oulu, Oulu, Finland; ^e^ Thule Institute, University of Oulu, Oulu, Finland; ^f^ School of Medical Sciences, Örebro University, Örebro, Sweden; ^g^ Audiological Research Centre, Örebro University Hospital, Örebro, Sweden

**Keywords:** Hearing loss, adult, Saami, indigenous

## Abstract

The Saami are the only indigenous population in Europe and their traditional living area is northern Scandinavia. Hearing impairment (HI) among Saami has not been studied before. The objective was to investigate the presence and type of HI among Saami adults, aged 49–77 years (median age 61 years), living in northern Finland. In addition, the presence of self-reported hearing difficulties, difficulties to hear in background noise and tinnitus were studied. An epidemiological, cross-sectional study encompassing a structured interview, otological examination and audiometry was performed. Bilateral HI was present in 42.9% of men and 29.4% of women, when HI was defined as a pure tone average (PTA) of at least 20 dB hearing level (HL) or more at the frequencies of 0.5, 1, 2 and 4 kHz. In one or both ears (worse ear hearing level, WEHL_0.5,1,2,4_≥20 dB HL) HI was present in 61.8% of men and 42.2% of women. Sensorineural high frequency hearing impairment was found to be most common. Nearly half (46.9%) of the study subjects reported hearing problems and more than half (55.6%) reported difficulties in following conversation in background noise. Measured HI and subjective hearing difficulties are common among the Saami adults. The healthcare personnel working in this area should be aware of the hearing problems of the Saami population.

**Abbreviations:** ARHI, Age-related hearing impairment; PTA, Pure tone average; HI, Hearing impairment; HL, Hearing level; BEHL, Better ear hearing level; WEHL, Worse ear hearing level; CI, Confidence interval

## Introduction

The Saami (or Sami) are the only indigenous population in Europe. According to genetic studies, the Saami are a genetically isolated population. It is thought that the Saami descended from a distinct sub-group of Europeans who settled the northern Fennoscandia, which means the northern areas of Finland, Norway, Sweden and the Kola peninsula of Russia, several thousands of years ago [1,2]. There is no close genetic relationship between the Saami and the Finns [3].

**Figure 1.**
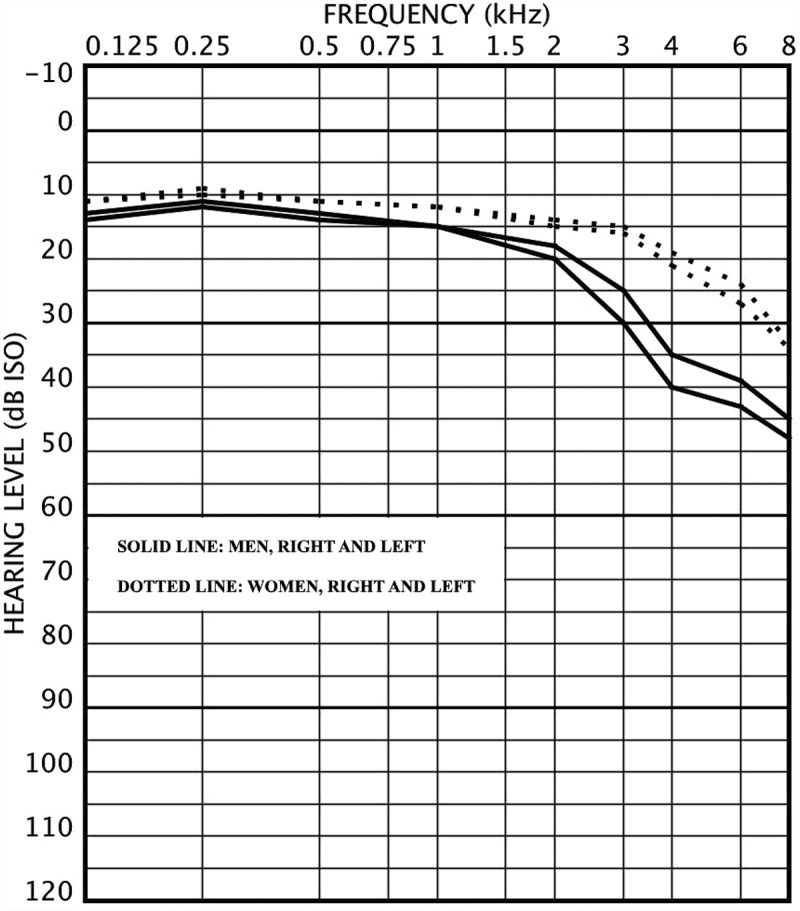
Audiogram for men (n=191) and women (n=139) aged 49–64 years with mean air conduction hearing levels.

**Figure 2.**
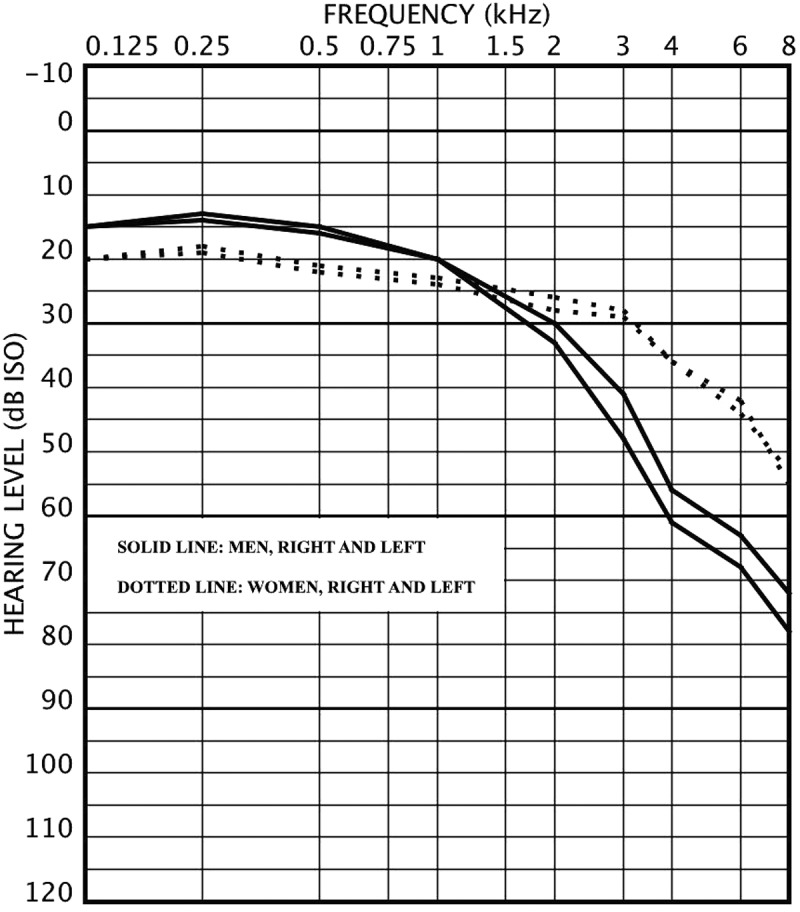
Audiogram for men (n=105) and women (n=79) aged 65–77 years with mean air conduction hearing levels.

In Finland, the definition of a Saami is laid down in the Act on the Saami Parliament and it is mainly based on the Saami language, like in Sweden and Norway. According to the definition, a Saami is a person who considers him- or herself a Saami, provided that this person has learnt Saami as his or her first language or has at least one parent or grandparent whose first language is Saami. Approximately 75,000–110,000 Saami live in the northern Fennoscandia, of which, as estimated, ~10,000 are in Finland, 60,000 in Norway, 36,000 in Sweden and 2,000 in Russia. In Finland, the preservation of Saami cultural heritage and language is guaranteed by law. Traditionally, the way of living among Saami has been reindeer herding, hunting, fishing and agriculture. In northern Finland, reindeer herding is still the most visible part of the Saami culture and also a way to earn a living in addition to tourism, forest work, service occupations and traditional handicrafts manufacturing. Nowadays, however, most of the Finnish Saami live outside the traditional living areas and have adapted a modernised lifestyle with other occupations (www.samediggi.fi).

Health-related research among the Saami has been increasing in recent years [4]. Compared to other circumpolar indigenous populations, the Saami have today better access to health services [5]. In Finland, as in Sweden and Norway, every citizen is entitled to get public health services, including hearing healthcare. In addition, according to the law, the Saami should get the health services provided by their own language. Anyhow, according to a Norwegian study, the Saami-speakers are less satisfied with the general practitioners’ services than the Norwegian speakers and especially with the physicians’ language skills [6]. To our knowledge, similar studies has not been made in Finland. The overall health status, life expectancy and mortality patterns of the Saami do not differ remarkably from non-indigenous residents of northern Fennoscandia [4,5]. Still there are concerns, as there is a high rate of suicides among the young reindeer herders, especially in Sweden [7].

Adult-onset HI, typically increasing with age and at first affecting the higher frequencies, is a common problem worldwide [8,9]. Earlier, HI has been reported to be common among other arctic indigenous populations in Canada and Greenland [10]. Moore [11] has studied hearing among native people (n=1897) in four regions in the western Canadian Arctic. HI was present among 57–63% of the subjects in one or both ears, depending of the region.

We found no previous epidemiological studies reporting hearing disorders among the Saami. The fact that HI has been reported to be a common condition among other indigenous populations gives a good reason to examine the hearing of the Saami. The aim of the present study was to investigate the presence and type of HI in an adult Saami population living in northern Finland. Furthermore, the presence of self-reported hearing difficulties and tinnitus were investigated.

## Subjects and methods

### Sampling

The subjects in the present study were collected in conjunction with the European ARHI Project (QLRT-2001–00331), a multi-centre study investigating environmental, medical and genetic factors contributing to age-related HI [12].

The study was focused to the traditional living areas of the Saami. The study area consisted of the three northernmost municipalities of Finland: Utsjoki, Enontekiö and Inari; as well as the Vuotso village of the municipality of Sodankylä. These arctic areas are rural and sparsely populated, where many Saami people make their living mainly by reindeer herding, tourism, service occupations and by making traditional handicrafts.

The inclusion criteria for the present study were ethnicity (Saami) and age. Individuals born in 1928–1954 were invited to the study, which was conducted during the years 2003–2006. The enrolment was made through the public population register in three steps. At first the study was geographically focused on the traditional living areas, with a good probability to identify Saami individuals. In the second step a letter of invitation was send to putative study subjects based on an expert evaluation of Saami communities. As the third step of the enrolment, the Saami identity was confirmed with each study subject in the interview. The letter of invitation was sent to 914 individuals. In addition, 11 subjects were recruited by their own contact or as the spouse of the original subject. Thus, the total number of the putative study subjects was 925, of whom 348 subjects did not participate in the study. The municipalities of Enontekiö and Utsjoki and the village of Vuotso were visited once. The biggest municipality, Inari, was visited three times. In all four municipalities, altogether 577 subjects (62.4%) participated in the study. Of these 577 participants, 63 were excluded at the study appointment. Of them, 32 subjects did not fulfil the ethnicity criterion and 16 subjects were excluded from the study because the reliability of the audiogram was uncertain, due to problems with calibration of the audiometer. The remaining 15 excluded subjects either declined to participate or were not able to give written informed consent because of disability or illness. Thus, altogether 514 Saami individuals (55.6% of invited subjects), 296 men (57.6%) and 218 women, were included in the study (Table 1). Mean age was 61 years (SD=7.4). Written informed consent was obtained from all subjects. The study was approved by the Finnish Advisory Board on Health Care Ethics. The subjects were not given any financial compensation for participating.

**Table 1.** Participation of the study subjects according to the study sites.

Study site	Enontekiö, n (%)	Inari, n (%)	Utsjoki, n (%)	Vuotso village of Sodankylä, n (%)	Total, n (%)
Invited, n	111 (12.0*^c^*)	457*^a^* (49.4*^c^*)	260 (28.1*^c^*)	97 (10.5*^c^*)	925
Participants	50 (45.0)	284 (62.1)	179 (68.8)	64 (66.0)	577 (62.4)
Excluded*^b^*	1 (2)	37 (13.0)	18 (10.1)	7 (10.9)	63 (10.9)
Included	49 (9.5^c^)	247 (48.1*^c^*)	161 (31.3*^c^*)	57 (11.1*^c^*)	514

*^a^* Including 11 subjects who participated by their own contact.

*^b^* Exclusion criteria: did not fulfil the ethnicity criterion (32 subjects), audiogram quality was poor due to problems with calibration of the audiometer (16 subjects), declined to participate or were not able to give written informed consent because of handicap or illness (15 subjects).

*^c^* Percentage of total.

The results of the present study were compared with a more urban Finnish population collected during 2003 and 2004 from the city of Oulu and its surroundings (hereafter referred as the Oulu study). Participants in the Oulu study were randomly selected from the population register on the basis of their living area and birth year. An invitation letter was mailed to 1,428 subjects aged 54–66 years, of whom 858 (60%) replied and a total of 850 subjects were eligible for the study, 383 men (45.1%) and 467 women (54.9%). Of the non-participants, 55.8% were men [13].

### Data collection

In Enontekiö, Inari and Utsjoki the study was conducted at the health centres. As there is no health centre in Vuotso, the local church hall was used to arrange the study appointments and audiological measurements. Only Enontekiö health centre provided a sound-insulated booth for the study purposes; in the other practices audiometry was performed in a quiet examination room.

A Madsen Midimate 602 (Otometrics, Denmark) clinical audiometer was used and it was calibrated according to ISO 389–1 (1998) and ISO 389–3 (1994). Supra-aural TDH-39 earphones with MX-41/AR cushions and a Radioear B-71 bone vibrator were used. Pure-tone air conduction thresholds (0.25, 0.5, 1, 2, 3, 4, 6 and 8 kHz) and bone conduction thresholds (0.25, 0.5, 1, 2 and 4 kHz) were measured by trained audiology assistants using the ascending method according to ISO 8253–1 (1989).

All the subjects underwent an otological examination made by an ear, nose and throat specialist (SH). The subjects completed a questionnaire, in which possible self-reported hearing problems were retrieved. The questionnaire was sent to the study subjects beforehand. All the replies in the questionnaire were checked during a structured interview at the study appointment by the study physician (SH). The study subjects’ hearing problems and tinnitus were retrieved by the following questions: “Do you have any difficulty with your hearing?”, “Do you find it very difficult to follow a conversation if there is background noise, e.g. TV, radio, children playing?” and “Nowadays, do you ever get noises in your head or ears (tinnitus) which usually last longer than 5 minutes?”

The study protocol in the Oulu study was the same, but the study was conducted at the audiological unit of Oulu University Hospital. The audiological tests were conducted in sound-isolated booths using Madsen Midimate 602 and Madsen Orbiter 922 (both from Otometrics, Denmark) clinical audiometers, which were calibrated according to ISO 389–1 (1998) and ISO 389–3 (1994) [13].

### Audiological criteria

For the presence of HI, the HI is defined as pure tone average (PTA) of at least 20 dB hearing level (HL) at the frequencies of 0.5, 1, 2 and 4 kHz in the better and in the worse ear (better ear hearing level, BEHL_0.5,1,2,4_≥20 dB HL or worse ear hearing level, WEHL_0.5,1,2,4_≥20 dB HL) as recommended by a European Union expert group in the HEAR project [14]. If pure-tone thresholds exceeded the maximum output of the audiometer, a value of 130 dB was used as the data entry according to the recommendation of the British Society of Audiology [15]. A conductive HI was defined as an air–bone gap averaged over 0.5, 1 and 2 kHz of ≥15 dB in one or both ears and a mixed HI as a conductive HI and mean bone conduction threshold over 0.5, 1 and 2 kHz ≥20 dB HL in one or both ears. HI was considered asymmetrical when the difference between the left and right ear air conduction thresholds was 20 dB or more for at least two frequencies out of 0.5, 1 and 2 kHz [14].

### Statistical analyses

Summary measurements for continuous variables are presented as means and standard deviations (SD) and as number of cases with percentage (%) for categorical variables. Pearson’s Chi-square test was used to compare the differences between men and women and Student’s t-test to compare PTA levels between the Saami and the Oulu populations. Analyses were performed using SPSS for Windows (IBM Corp. Released 2012. IBM SPSS Statistics for Windows, Version 21.0. IBM Corp., Armonk, NY).

## Results

### Audiogram and questionnaire data

The mean age was 61.1 years (SD=7.7) for men and 61.4 years (SD=7.3) for women (p=0.66). Hearing impairments and self-reported hearing difficulties are presented in Table 2. In general, HI was more common among men than among women, as were self-reported hearing difficulties. No significant differences were found between the four study sites. Audiograms drawn with mean hearing levels at different frequencies are presented in Figure 1 and 2, for two age groups: 64 years or younger and 65 years or older. Right ear hearing levels were slightly better than those of the left ears in both age groups and genders and the difference was greater in men at the higher frequencies. In the younger age group, women had better hearing than men at all frequencies. In the older age group, men had better hearing at lower frequencies up to 1 kHz and women have better hearing at higher frequencies (>1 kHz). When analysing the pure tone averages (PTA) at low (0.125, 0.25 and 0.5 kHz), mid (0.5, 1, 2 and 4 kHz) and high (4, 6 and 8 kHz) frequency areas, men had significantly poorer hearing at mid and high frequencies. At low frequencies there were no significant difference between genders. PTA figures in different frequency areas and the comparison with the Oulu study [13] are shown in Table 3. The PTA figures of the Oulu population are lower (better) in all frequencies and in both genders.

**Table 2.** The presence of hearing impairment (HI) and self-reported hearing problems according to gender and among all subjects. Hearing impairment defined as better ear hearing level (BEHL) or worse ear hearing level (WEHL) of 20 dB or more at the 0.5–4 kHz frequency range. Pearson Chi-square test was applied for gender differences.

Hearing impairment or hearing problem	Men (296), n (%)	Women (218), n (%)	p-value	All (514), n (%)
Hearing impairment				
BEHL_0.5–4 kHz_≥20 dB HL	127 (42.9)	64 (29.4)	0.002	191 (37.2)
WEHL_0.5–4 kHz_≥20 dB HL	183 (61.8)	92 (42.2)	<0.001	275 (53.5)
Any self-reported hearing difficulties	149 (50.3)	92 (42.2)	0.074	241 (46.9)
Difficulties to follow conversation in background noise	187 (63.2)	99 (45.4)	<0.001	286 (55.6)
Tinnitus	99 (33.4)	60 (27.5)	0.15	159 (30.9)

**Table 3.** The comparison of pure-tone averages and standard deviations (in brackets) between the present study (Saami) and a previously published population-based study (Oulu) of Hannula et al. [13]. The figures are shown separately for the different frequency areas, for both genders and for both ears. T-test applied for differences between the two study populations.

		Men			Women			All		
Frequency area	Ear	Saami (n=296)	Oulu (n=383)	p-value	Saami (n=218)	Oulu (n=467)	p-value	Saami (n=514)	Oulu (n=850)	p-value
Low 0.125, 0.25 and 0.5 kHz	R	14.0 (18.0)	12.2 (13.1)	0.016	14.0 (16.3)	11.6 (10.6)	0.024	14.0 (17.3)	11.9 (11.8)	0.007
	L	13.1 (12.4)	11.0 (11.0)	0.124	13.8 (14.6)	11.1 (11.2)	0.007	13.4 (13.4)	11.0 (11.1)	<0.001
Mid 0.5, 1, 2 and 4 kHz	R	24.1 (18.8)	21.0 (15.3)	0.008	18.9 (16.9)	15.8 (12.0)	<0.001	21.9 (18.2)	18.2 (13.8)	<0.001
	L	25.8 (16.5)	21.8 (13.9)	<0.001	19.1 (15.7)	15.6 (11.4)	<0.001	22.9 (16.5)	18.4 (13.0)	<0.001
High 4, 6 and 8 kHz	R	48.2 (26.9)	44.1 (22.6)	0.029	32.2 (22.6)	29.9 (16.5)	0.126	41.4 (26.4)	36.3 (20.7)	<0.001
	L	52.6 (26.8)	49.9 (22.6)	0.153	33.4 (21.3)	33.1 (18.1)	0.867	44.5 (26.4)	40.7 (21.9)	0.005

When comparing self-reported hearing difficulty to the measured HI, we found that 54.8% of the subjects who reported difficulties in hearing had measured bilateral HI (BEHL_0.5,1,2,4_≥20 dB HL) and 76.3% had measured HI in one or both ears. Of the subjects, 30.9% with bilateral HI and 33.1% of those with at least unilateral HI did not report any hearing difficulties.

### Clinical examination of the ears

Ear canals were normal in 509 (99%) of the study subjects. Four subjects had narrowed ear canals on both sides and one subject showed infection in the left ear canal. Tympanic membranes were normal in 487 (94.7%) subjects on the right side and in 491 (95.5%) subjects on the left side. Abnormalities were present in 34 subjects (6.6%). Fifteen subjects had abnormalities in both tympanic membranes, 11 subjects only on the right side and eight subjects only on the left side. The abnormalities can be listed as follows: perforation (eight subjects), sequel after previous ear operation (nine subjects), poor mobility of the tympanic membrane (six subjects), atrophic tympanic membrane (nine subjects) or adhesions of tympanic membrane (seven subjects).

### Type of HI

Of all hearing impaired subjects (WEHL_0.5,1,2,4_≥20 dB HL), 91.9% (n=252) had a sensorineural HI and 8.1% (n=23) showed a conductive or mixed type HI. Only eight subjects (1.6%) had a pure conductive HI in one or both ears. Among all study participants, 4.3% had a conductive or a mixed type HI. The type of HI could not be defined for four subjects because of missing bone conduction thresholds. Forty-eight subjects (17.5% of all subjects with HI) had asymmetrical HI, of them 14 had a conductive or mixed type HI and 33 subjects had asymmetrical sensorineural HI.

## Discussion

Hearing impairment was a common finding in our study population. To our knowledge, hearing among Saami has not been studied before. It is remarkable that more than 60% of the Saami men and more than 40% of the Saami women had HI in one or both ears. We did not find any recent studies concerning hearing problems among indigenous arctic populations for comparison. More than 30 years ago, high prevalence of hearing loss was reported in the Eskimo population (n=3574) of Baffin Zone, Canada [16]. In that study, most of the study population (74.8%) were under 22 years. Among adults, high prevalence of sensorineural HI was perceived, especially in males. Similar results were found in the study of Counter and Klareskow [17] in Polar Eskimos of Northwest Greenland, aged 6–80 years. In the latter, the study population was rather small (75 men and 43 women) and, because of the time gap and possible changes in the lifestyle, these results are not fully comparable to ours.

For comparison, we have used data from the Oulu study, in which the mean age, age distribution and the time period when the data is collected, are nearly the same as in our study [13]. In the Oulu population, the overall prevalence of HI was 26.7% (36.8% among men and 18.4% among women). This is remarkably lower than in the Saami population and a comparison of the audiogram data (Table 3) shows that the differences are significant and most clear in the mid frequency area. In the National Study of Hearing (NHS) conducted by Davis *et al*. [18], the PTA of 51–60 year old adults in the middle frequencies (0.5–4 kHz) is 20.1 dB for both ears, which are slightly higher than in the Oulu study, but lower than in the Saami. The type of HI among Saami was mostly sensorineural, while only 8.1% of all hearing impaired subjects had conductive or mixed HI, which is 4.3% of the whole study population. This proportion does not differ from previous, large population-based studies. In the study conducted in Oulu and surroundings, the proportion of conductive or mixed HI was 6.1% [13], in the Epidemiology of Hearing Loss Study, the proportion of conductive HI was 8.1% [8] and in the NHS, the prevalence of conductive HI was 3.4% in 51–60 year old adults [18].

Men have generally poorer hearing than women [8,19]. In the current study population, in the younger age group men have poorer hearing in all frequencies. In the older age group, the Saami men have better hearing at the low frequencies than the Saami women. A similar finding has been reported previously by some other researchers, i.e. in Sweden [20] and in the US [21,22]. However, such finding could not be seen in the population-based study of Hannula *et al*. [13].

Considering the fact that HI among Saami adults has not been studied before, the study population of 514 subjects is relatively large and represents approximately 5% of all Finnish Saami. Sampling of the study population was challenging. No official register for the Saami people is available for research purposes and it is forbidden by law to keep any registers based on ethnicity. Furthermore, the Saami ethnicity, as defined according to Finnish legislation, is not always quite clear. This challenge is described also in a Norwegian study concerning the health of Saami [23]. In order to control the possible sampling bias, the sampling was conducted in three phases, Saami expertise was used to identify Saami individuals and the Saami identity was confirmed with each study subject in the interview. The definition is cultural and based on self-report, but still the Saami seem to be a separate group also genetically, which increases the reliability of the sampling [24,25]. About 60% of invited subjects participated in the study and, unfortunately, we do not have further analysis of non-participants. It is possible that those subjects who have problems with hearing are more eager to participate [26]. In addition, only the minority of Finnish Saami live in the study’s geographical area.

Audiometry was performed mostly in quiet examination rooms, as sound-isolated booths were not available in all study sites, which may have some influence on the results. However, Maclennan-Smith *et al*. [27] have compared the hearing test results made in a sound-isolated booth or in a natural environment and found a 95% correspondence in air conduction hearing thresholds between these two test environments. Hence, the influence of the different test environment is not considered to be significant.

The results of the present study can be used in planning hearing healthcare services in northern Finland. Our results can rather reliably be applied for the Saami living in the northern areas of Norway and Sweden, considering the similarities in the living environment and the healthcare systems. Collaboration between the Nordic Countries would be useful in planning and organising healthcare in the circumpolar area and among indigenous people [4,5]. Further, our study provides a practical message to the healthcare personnel: they should be aware of the frequent hearing problems of the Saami. The symptoms of HI should be actively inquired and, when needed, the patients should be referred to an audiological assessment. The aetiology (genetic and/or acquired conditions) behind HI among the Saami will be a topic for further research.

## Conclusion

Hearing impairment among Saami has not been studied before. In this study, both measured HI and self-reported hearing difficulties were common among Saami adults living in northern Finland. This should be taken into account when planning health services for the Saami and the healthcare personnel working in this area should be aware of the hearing problems of the Saami population.
